# Afterglow Carbon Dots: From Fundamentals to Applications

**DOI:** 10.34133/2021/6098925

**Published:** 2021-02-04

**Authors:** Chenxi Peng, Xue Chen, Meiling Chen, Shenci Lu, Yu Wang, Suli Wu, Xiaowang Liu, Wei Huang

**Affiliations:** ^1^ Frontiers Science Center for Flexible Electronics (FSCFE), MIIT Key Laboratory of Flexible Electronics (KLoFE), Shaanxi Key Laboratory of Flexible Electronics, Xi’an Key Laboratory of Flexible Electronics, Xi’an Key Laboratory of Biomedical Materials & Engineering, Xi’an Institute of Flexible Electronics, Institute of Flexible Electronics (IFE), Northwestern Polytechnical University, Xi’an, 710072 Shaanxi, China; ^2^ International Collaborative Laboratory of 2D Materials for Optoelectronics Science and Technology of Ministry of Education, Institute of Microscale OptoelectronicsChina; ^3^ State Key Laboratory of Fine Chemicals, Dalian University of Technology, 2nd Linggong Road, Dalian 116024, China; ^4^ Key Laboratory for Organic Electronics and Information Displays & Institute of Advanced Materials (IAM), Nanjing University of Posts & Telecommunications, 9 Wenyuan Road, Nanjing, China; ^5^ Key Laboratory of Flexible Electronics (KLOFE) and Institute of Advanced Materials (IAM), Nanjing Tech University (NanjingTech), 30 South Puzhu Road, Nanjing 211816, China

## Abstract

The ability of carbon dots (CDs) to emit afterglow emission in addition to fluorescence in response to UV-to-visible excitation allows them to be a new class of luminescent materials. When compared with traditional organic or inorganic afterglow materials, CDs have a set of advantages, including small size, ease of synthesis, and absence of highly toxic metal ions. In addition, high dependence of their afterglow color output on temperature, excitation wavelength, and aggregation degrees adds remarkable flexibility in the creation of multimode luminescence of CDs without the need for changing their intrinsic attributes. These characteristics make CDs particularly attractive in the fields of sensing, anticounterfeiting, and data encryption. In this review, we first describe the general attributes of afterglow CDs and their fundamental afterglow mechanism. We then highlight recent strategic advances in the generation or activation of the afterglow luminescence of CDs. Considerable emphasis is placed on the summarization of their emergent afterglow properties in response to external stimulation. We further highlight the emerging applications of afterglow CDs on the basis of their unique optical features and present the key challenges needed to be addressed before the realization of their full practical utility.

## 1. Introduction

Afterglow is an interesting optical phenomenon in which a substance releases accumulated energy in the form of photons after removal of the excitation source [1]. In comparison to fluorescence that has a spontaneous emission upon excitation (within 10 ns), afterglow exhibits lifetime longer than 0.1 ms [2, 3]. In some cases, efficient release of the stored energy needs additional excitation such as thermal, rendering the feasibility to achieve stimulus-responsive long-lived emissions [4]. The ability of afterglow materials to emit long-lived emissions allows them to be easily distinguished from background fluorescence and to find widespread applications in the fields of lighting, bioimaging, anticounterfeiting, and optical recording [5–9].

In addition to traditional inorganic afterglow materials, organic afterglow counterparts have attracted more and more attention due to the fact that their afterglow attributes, such as wavelength, lifetime, and quantum yield, can be facilely tuned via molecule or crystal structure engineering [10]. Modern research of organic afterglow starts in 2007 by Zhang et al., who reported the observation of a long lifetime from a composite of difluoroboron dibenzoylmethane and poly(lactic acid) [11–15]. Organic afterglow materials mainly include organometallic complexes, metal-free crystalline organic compounds, polymers, metal-organic frameworks (MOFs), and carbon dots (CDs) [16–20]. When compared with other organic afterglow phosphors, CDs exhibit inherent advantages in their practical utility: (i) their main component is carbon, showing less potential toxicity and environmental concerns [21, 22]; (ii) their synthetic procedure is simple, without the need for tedious protocols and complex experimental setup; (iii) their size is small, thus allowing them to find applications in newly emerged nanotechnologies, such as bioimaging and printable inking [23, 24]; and (iv) their afterglow feature is tunable, permitting the creation of multiple long-lived color codes for multiplexing and information storage.

**Figure 1:**
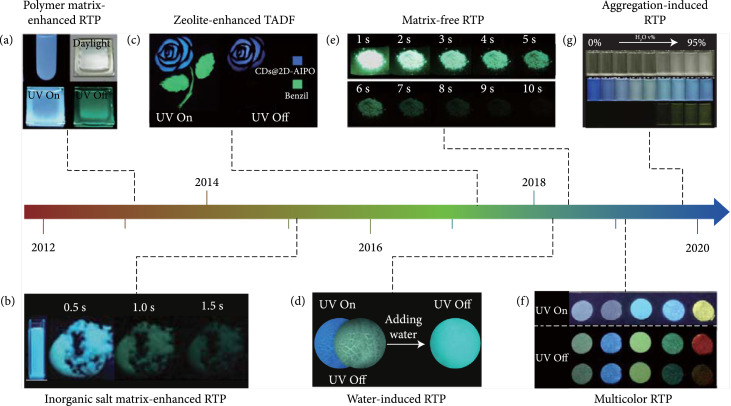
Selective milestones in the development of afterglow CDs in the last decade. (a) Deng et al. first reported the observation of RTP of CDs after being incorporated in a PVA matrix in 2013 (adapted and copyright permission [25], Royal Society of Chemistry). (b) Dong et al. observed a similar role of inorganic salts, such as KAl(SO_4_)_2_·x(H_2_O), in the activation of the RTP of CDs in 2015 (adapted and copyright permission [27], Royal Society of Chemistry). (c) Liu et al. demonstrated the observation of long-lived thermally activated delayed fluorescence (TADF) of CDs after being encapsulated into zeolite matrices via a general “dots-in-zeolites” strategy in 2017 (adapted and copyright permission [28], American Association for Advancement of Science). (d) Li and coworkers described the water-induced RTP of CDs due to the formation of hydrogen bonding networks between CDs and cyanuric acid in 2018 (adapted and copyright permission [29], Nature Publishing Group). (e) Jiang et al.’s group reported the preparation of self-protected RTP CDs via microwave-assisted heating of a mixture of EAM and phosphoric acid in 2018 (adapted and copyright permission [30], Wiley-VCH Verlag GmbH & Co. KGaA). (f) Li et al. made the demonstration of achieving multiple RTP color output of CDs via encapsulation of CDs with different compositions into boric acid matrix in 2019 (adapted and copyright permission [39], Wiley-VCH Verlag GmbH & Co. KGaA). (g) Jiang and coworkers reported the observation of aggregation-induced RTP of CDs with enhancing the water fraction in a THF dispersion of CDs at room temperature in 2019 (adapted and copyright permission [40], Wiley-VCH Verlag GmbH & Co. KGaA).

Since the pioneering report in 2013 on room temperature phosphorescence (RTP) of CD-doped poly(vinyl alcohol) (PVA) composites by Deng et al.’s group [25], exploration of the synthetic strategies towards afterglow CDs and understanding of their underlying afterglow mechanism have been the focus of a growing body of research in the field of optical materials science [26]. Considerable advances have been achieved in terms of stabilizing the triplet excited species of CDs [27], tuning afterglow luminescence of CDs, and expanding their applications on the basis of their unique optical features (Figure 1) [28–30]. Recent efforts have been devoted to understanding the emergent afterglow nature of CDs, such as excitation-dependent afterglow, temperature-responsive afterglow, and aggregation-induced RTP.

Owing to the remarkable advances, a review work that provides a comprehensive summarization in the past decade is highly necessary. In this review, we start from a fundamental introduction of afterglow CDs, including physical characteristics and afterglow mechanism. Next, we describe a toolbox for activating the afterglow luminescence of CDs with an emphasis on the methods with the ability to tune their afterglow lifetime and color output. Parallel efforts are devoted to highlighting the emergent attributes of the afterglow luminescence of CDs. Then, we place our focus on the presentation of recent applications of afterglow CDs, ranging from sensing, bioimaging to anticounterfeiting, and data encryption. In the last section, we discuss the challenges and opportunities for afterglow CDs during the realization of their practical utility.

## 2. Fundamental Aspects of Afterglow CDs

CDs are an important member of the big family of nanosized carbon materials. On the basis of their detailed structural characteristics, they are also referred to as carbon nanoparticles, carbon nanodots, carbonized polymer dots, graphene quantum dots, and polymer dots [31–33]. Note that even with the assistance of surface activation, only a small fraction of CDs are found to have the ability to emit long-lived emissions upon excitation. The detailed structural features of afterglow CDs are discussed in this section, including size, composition, crystallinity, and surface moiety. These intrinsic features act together to produce unique afterglow attributes for CDs.

### 2.1. Structure

**Figure 2:**
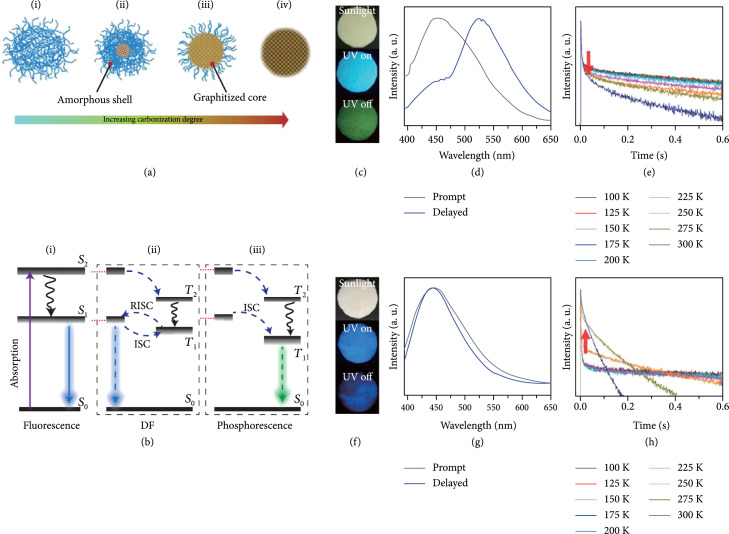
(a) Schematic representation of four stages in the preparation of CDs via a high-temperature carbonization strategy. (i) Formation of cross-linked amorphous CDs. (ii and iii) Formation of CDs with a controlled graphitized core and an amorphous shell. (iv) Formation of over-carbonized CDs. This kind of CDs is produced at high temperatures, endowing them with poor surface chemistry and inability to luminesce upon excitation (adapted and copyright permission [35], American Chemical Society). (b) Schematic energy level diagrams showing the generation of luminescence of CDs, including fluorescence, DF, and phosphorescence. The afterglow luminescence mechanism for CDs mainly comprises TADF, phosphorescence, and a combination of the two. (c) Photographs of CDs@SBT-1 under sunlight, UV-on, and UV-off states. (d) The corresponding emission profiles of fluorescence and phosphorescence. (e) Temperature-dependent decay behaviors of the phosphorescence (at 525 nm). (f) Photographs of CDs@SBT-2 under sunlight, UV-on, and UV-off states. (g) The corresponding emission profiles of fluorescence and DF. (h) Temperature-dependent decay behaviors of the DF (at 440 nm) (adapted and copyright permission [57] (c–h), American Chemical Society).

At present, the majority of afterglow CDs are derived from high-temperature carbonization of molecule or polymer precursors in solution or solid phases [34]. Carbonization reactions typically proceed through dehydration of the functional groups of the precursors, which can be classified into four stages (Figure 2(a)) [35]. In the early stage, dehydration reactions lead to the formation of cross-linked amorphous carbon polymers, sometimes with emissive characteristics but no nonconjugated system (Figure 2(a) (i)). This phenomenon is known as a cross-link-enhanced emission effect [36, 37]. However, such amorphous CDs usually show the inability to emit long-lived emissions even after being subjected to surface treatment because the excited triplet species are easily deexcited by the vibration of the matrix [38]. With prolonging reaction time or enhancing reaction temperature, cross-linking degrees within the amorphous CDs are significantly improved, leading to the emergence of graphitized cores. The presence of graphitized cores within CDs not only adds considerable flexibility in tuning their fluorescence via control over the size of the conjugated cores (Figure 2(a) (ii and iii)) but also leads to remarkable enhancement in their structural rigidity. The latter feature and highly cross-linked polymer-like surface layer can largely limit the vibration freedom of subfluorophores, such as C=O, C=N, and N=O, allowing for the generation of afterglow luminescence without the need for surface activation. However, the introduction of additional surface stabilization matrix is necessary in most cases to further enhance the stability of the excited triplet species and to result in the generation of improved afterglow luminescence. Continued carbonization gives rise to the formation of nonemissive CDs due to a combination of small energy gaps between excited and ground singlet states and the poor surface chemistry (Figure 2(a) (iv)).

These structural features suggest that afterglow CDs are essentially in analogy to quantum dots, which have a crystalline core and an organic ligand shell. A slight difference lies in the fact that the interaction between the surface moieties and core components for CDs shows covalent nature. The reported graphitized cores are in the range of 1 to 10 nm and show a limited impact on the afterglow emission wavelength of CDs (Table 1). While the luminescent properties of CDs are determined by a combination of a small-sized conjugated domain, surface defects, quasimolecules, and subfluorophores [53, 54], only subfluorophore components seem to make a contribution to the afterglow luminescence. This is because the n→π∗ transitions of these groups permit efficient spin-orbit coupling, facilitating the generation of excited triplet species that are essential for the production of long-lived emissions for CDs.

**Table 1:** The dependence of afterglow attributes of CDs on their physic parameters and the synthetic conditions.

Precursors	T (°C)	Elements	Carbonization method	Size (nm)	Afterglow color	Lifetime (s)	Remarks	Refs
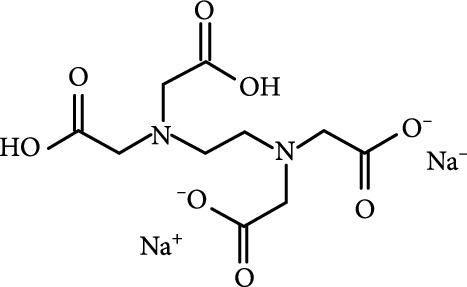	400	C, N, O	Solid	<5	Green (500 nm)	0.380	CDs@PVA	[25]
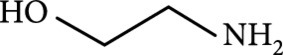	150	C, N, O	Solution	3	Green (500 nm)	0.655	CDs@KAl(SO_4_)_2_·x(H_2_O)	[27]
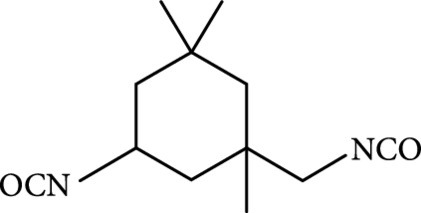	250	C, N, O	Solution	3.5	Green (500 nm)	0.005	CDs@polyurethane	[41]
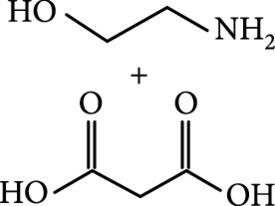	130	C, N, O	Solution	5	Green 525 nm	1.8^a^	CDs@SiO_2_	[42]
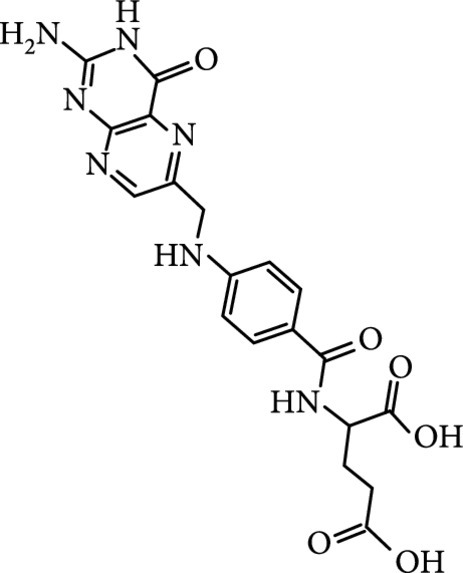	260	C, N, O	Solution	3.41	Green (490 nm)	0.93	CDs-biuret@urea	[43]
	255	C, N, O	Solution	2.0	Blue (430 nm)^b^	1.11	CDs-biuret@urea	[44]
					Green (500 nm)^c^	0.53		
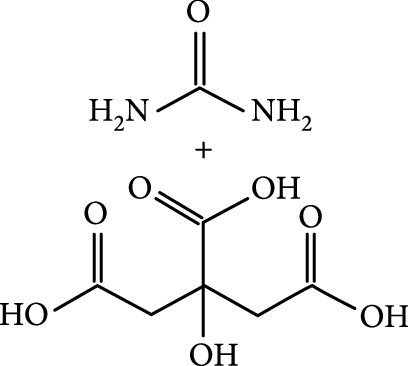	250	C, N, O	Solution	4.8	Blue (480 nm)	0.687^d^	CDs@cyanuric acid	[29]
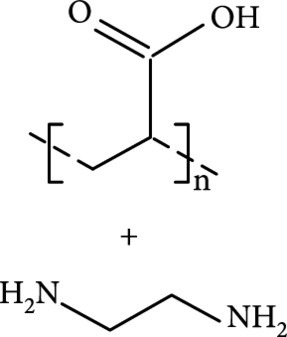	200	C, N, O	Solution	5.4	Blue-green (494 nm)	0.658	CD powder	[45]
	200	C, N, O	Solution	3.1	Green (520 nm)	1.64	CDs@SiO_2_	[46]
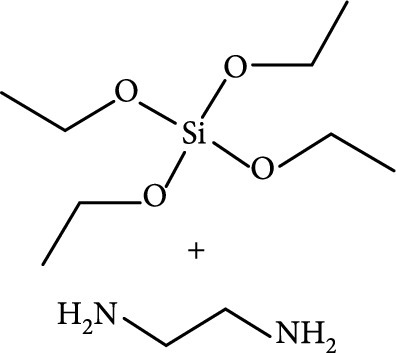	240	C, N, O	Solution	5.0	Green (520 nm)	1.26	CDs@SiO_2_	[47]
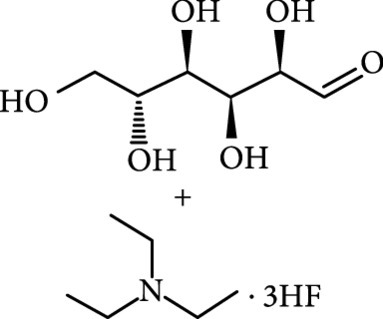	200	C, N, O, F	Solution	4.75	Green (455 nm)	1.045	CDs	[48]
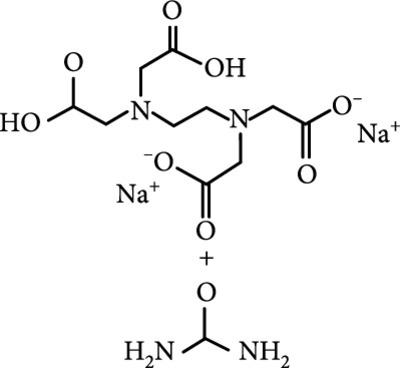	200	C, N, O	Solution	4.1	Green (529 nm)	0.269	CD powder	[49]
					Green (529 nm)	0.664	CDs@melamine	
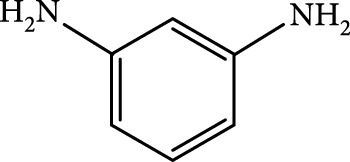	180	C, N, O	Solution	—	Green (506 nm)	0.456	CDs@PVA	[50]
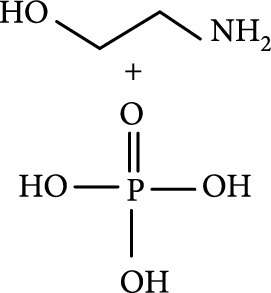	—	C, N, O, P	Solution	3.4	Green (535 nm)	1.46	CD powder	[30]
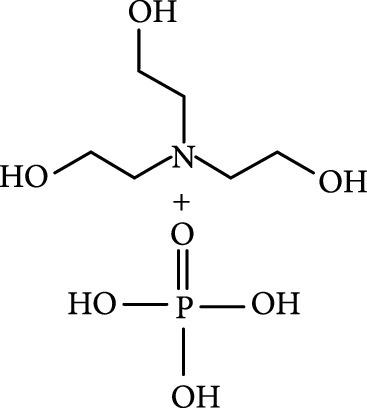	—	C, N, O, P	Solution	1.83	Green (518 nm)	0.82	CDs on paper	[51]
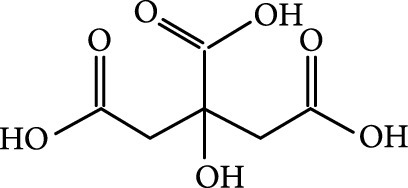	200	C, O	Solution	1.4	Green (530 nm)	1.6	CDs@boric acid	[39]
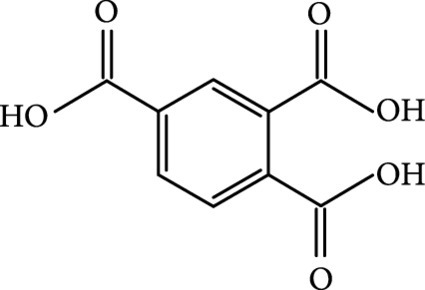	260	C, O	Solution	4.3	Yellow (560 nm)	0.184	CD powder	[40]
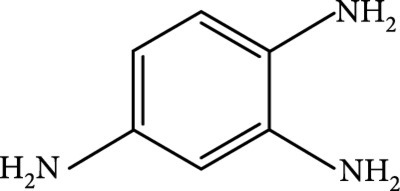	350	C, N, O	Solid	2.33	Green (566 nm)	0.701	CDs@molten salt	[52]
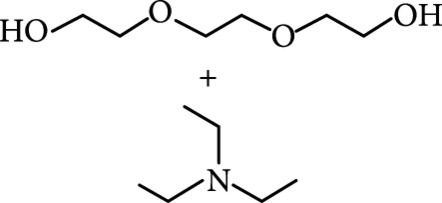	180	C, N, O	Solution	3.7	Blue (430 nm)	0.350	CDs@zeolite	[28]
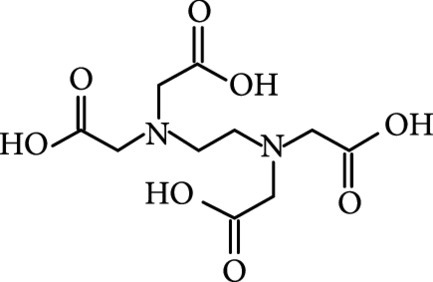	350	C, N, O	Solid	—	Green (520 nm)	1.64	CDs@SiO_2_	[46]
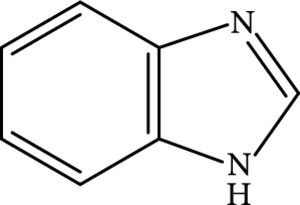	350	C, N, O, F	Solid	—	Green (455 nm)	1.045	CDs	[48]

^a^The lifetime was obtained via a single exponential fitting. ^b^Excitation at 254 nm. ^c^Excitation at 365 nm. ^d^Lifetime measurement was conducted in 70% water.

### 2.2. Composition

To facilitate the production of the above-mentioned subfluorophores, precursors should be carefully selected before the preparation of CDs via carbonization (Table 1). Heteroatom-containing precursors are highly desirable on the consideration of realizing heteroatom doping to promote the occurrence of intersystem crossing (ISC) [40]. For example, ethylenediaminetetraacetic acid disodium salt (EDTA·2Na), ethanolamine (EAM), ethylenediamine (EDA), and urea have proven effective as useful precursors for N doping [25, 49], while phosphoric acid shows great promise as a precursor for P doping. Notably, halogen doping also shows the ability to enhance spin-orbit coupling to boost ISC from the lowest singlet excited state ($S_{1}$) to the lowest triplet excited states ($T_{1}$) [55]. This argument has been verified by Knoblauch and coworkers, who first reported the observation of enhanced phosphoresce of CDs after being subjected to surface bromination [56]. However, a similar iodization treatment showed the inability to afford phosphorescence enhancement possibly due to a low iodine doping efficiency as a result of the weak bonding between carbon and iodine.

The detailed photoluminescence processes of CDs have been shown in Figure 2(b), including fluorescence delayed fluorescence and phosphorescence. It is important to note that the photophysical mechanisms are essentially similar to those previous findings in scintillator materials upon excitation with high-energy X-ray beams [58, 59]. In this case, electrons that are promoted to excited singlet states (such as $S_{2}$) upon UV light excitation are allowed to relax to the lowest $S_{1}$ and further to the singlet ground state ($S_{0}$) with the generation of fluorescence (Figure 2(b) (i)). In addition to direct relaxation between singlet states, the S_2_ and S_1_ electrons have a considerable possibility to across to the triplet excited states ($T_{2}$ or $T_{1}$) via the ISC process. The subsequent reverse intersystem crossing (RISC) of electrons from $T_{1}$ to $S_{1}$ followed by radiative $S_{1}$-to-$S_{0}$ transition allows the production of delayed fluorescence (DF) (Figure 2(b) (ii)). For DF to efficiently proceed, additional thermal activation is usually needed to promote the transition of electrons from $T_{1}$ to $S_{1}$, enabling the generation of thermally activated delayed fluorescence (TADF). Owing to its strong ability to harvest the excitation energy from the triplet excited states, DF materials usually exhibit high quantum efficiency [28]. Alternatively, phosphorescence can be generated from CDs via direct radiative deactivation from the excited electrons from the $T_{1}$ to $S_{0}$ state (Figure 2(b) (iii)). Of particular note is the possibility of the occurrence of ISC processes from multiple singlet excited states, especially in the cases of CD excitation with high energy. This character allows the intensity of phosphorescence to fluorescence to show high dependence on excitation energy [60]. Owing to the spin-forbidden nature of the triplet-to-singlet transition, both DF and phosphorescence have a long lifetime and account for the afterglow luminescence of CDs.

The luminescence mechanism suggests that the Stokes shift of phosphorescence is larger than that of DF [57]. For example, there is a shift of up to 72 nm between fluorescence and phosphorescence of 1-CDs@zinc aluminophosphate SBT zeolite (CDs@SBT-1) (Figures 2(c) and 2(d)) and no emission wavelength difference between fluorescence and DF of 2-CDs@zinc aluminophosphate SBT zeolite (CDs@SBT-2) (Figures 2(f) and 2(g)). Note that 1-CDs and 2-CDs were, respectively, derived from the carbonization of 4-(2-aminoethyl)-morpholine and 4,7,10-trioxa-1,13-tridecanediamine at 170°C for 7 days during the preparation of SBT, suggesting the possibility of tuning afterglow features of CDs via the selection of precursors in their preparation.

The underlying afterglow mechanism can be readily differentiated by temperature-dependent emission and decay profile analysis (Figures 2(e) and 2(h)) [57]. With decreasing experiment temperatures, vibrational motion and nonradiative transitions are gradually reduced. This effect renders phosphorescence with an increased trend in the emission intensity and a prolonged lifetime. Contrary to the case of phosphorescence, the elevation of experiment temperatures in most cases plays a positive role in enhancing DF intensity with a prolonged lifetime as a result of thermal-induced improved efficiency of the RISC from $T_{1}$ to $S_{1}$. However, when the experiment temperature is higher than a critical value, both emission intensity and lifetime of CDs will be decreased due to the occurrence of the thermal-induced nonradiative deactivation.

In essence, the emission wavelength difference between phosphorescence and DF originates from the energy gap ($\Delta E_{\text{ST}}$) between $S_{1}$ and $T_{1}$. The value can be estimated by

(1)ΔEST=ES1−ET1,

where $E_{S_{1}}$_1_ and $E_{T_{1}}$ represent the energy at the peaks of the steady-state and delayed photoluminescence profiles at 77 K, respectively. When $\Delta E_{\text{ST}}$ is generally smaller than 0.2 eV, DF usually becomes dominant as a result of the thermal effect. Notably, when $\Delta E_{\text{ST}}$ is slightly higher than the thermal-activatable energy, a combination of TRP and TADF usually appears, as characterized by the observation of the main band emission and a shoulder emission in the off-gated spectrum, and the peak of the shoulder emission is similar to that of the prompt fluorescence.

Owing to the presence of a wide range of chemical environments for subfluorophores and the multiple afterglow mechanisms, the afterglow decays of CDs usually show complicated exponential behaviors. As a result, the afterglow nature of CDs is often evaluated by an average lifetime ($\tau_{\text{a}}$) via

(2)τa=∑αiτi2∑αiτi,

where $\alpha_{i}$ and $\tau_{i}$ represent the portion and lifetime contribution of each component, respectively. Both $\alpha_{i}$ and $\tau_{i}$ can be obtained by fitting measured decay curves.

Notably, previous findings (Table 1) also suggest that the majority of the reported matrix-protected or matrix-free CDs show green afterglow luminescence with a band peak of less than 550 nm. The energy gap between $S_{1}$ and $T_{1}$ allows to be reduced by increasing the density of oxygen-containing groups on the surface of CDs. This fact implies the feasibility of tuning the afterglow luminescence of CDs to the red or near-infrared region via enhancing carbonization temperatures in the synthesis [61].

## 3. Activation of Afterglow Luminescence of CDs

As previously mentioned, the afterglow luminescence of CDs mainly originates from the n→π∗ transition from their surface subfluorophores. The main design principle to produce strong afterglow luminescence for CDs is governed by the consideration of a combination of enhancing their ISC efficiency and stabilizing their excited triplet states. Heteroatom doping has proven effective in promoting the efficiency of ISC by enhancing spin-orbit coupling. Stabilization of the excited triplet species for CDs necessitates fixation of the surface luminogens to suppress their vibration and rotation via either noncovalent or covalent bonding. The process used to protect the excited triplet states to enable the generation of afterglow luminescence of CDs is known as activation. According to the details in the experiments, activation strategies toward afterglow CDs can be divided into two-step, one-step, and self-activation methods.

### 3.1. Two-Step Activation Methods

Two-step activation methods involve the preparation of CDs in the first step followed by encapsulation of the CDs into different kinds of matrices. Such activation treatment imparts CDs with an afterglow feature in addition to fluorescence. According to the difference in the interactions between CDs and matrixes, two-step activation methods can be further divided into three strategies: (i) hydrogen bonding activation, (ii) complexing bonding activation, and (iii) covalent bonding activation.

#### 3.1.1. Hydrogen Bonding Activation

**Figure 3:**
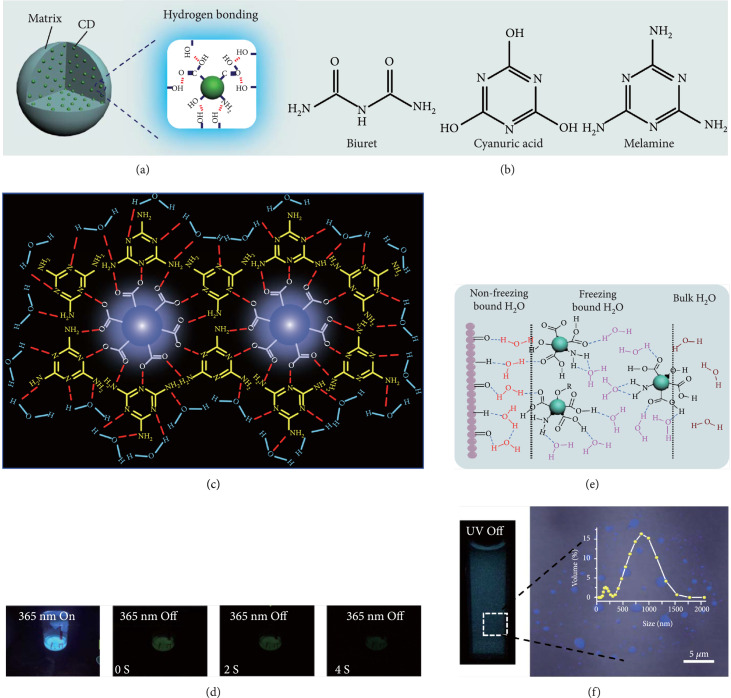
(a) Schematic representation of activation of the afterglow luminescence of CDs through hydrogen bonding. (b) Small molecules used to construct hydrogen bonding for activating the afterglow luminescence of CDs. (c) Schematic illustration of the formation of hydrogen bonding network between CDs and melamine to activate the afterglow luminescence of CDs. (d) Photographs of CDs@melamine powder under UV on and off states with time (adapted and copyright permission [49] (c, d), American Chemical Society). (e) Schematic illustration of the interactions among the CDs, CA particles, and water molecules. (f) Photograph (left) of the CD@CA suspension upon turn-off of UV light and the corresponding fluorescence image (right). Note that the fluorescence image was obtained by confocal laser scanning microscopy. The inset shows the size distribution of CD@CA obtained via dynamic light scattering (adapted and copyright permission [29] (e, f), Nature Publishing Group).

Owing to the fact that there are abundant subfluorophores on the surface of CDs, encapsulation of preprepared CDs into matrices with the promise to form hydrogen bonding allows the activation of their afterglow luminescence (Figure 3(a)). Since the first report on polymer-activated afterglow luminescence of CDs in 2013, a variety of polymer matrices, including PVA, polyacrylic acid (PAA), polyacrylamide, and polyurethane, have proven effective in stabilizing the excited triplet states of CDs, thereby rendering the CD-doped polymer composites with afterglow luminescence [25, 41, 50, 62–67]. Obviously, the underlying excited triplet stabilization mechanism is due to the formation of abundant hydrogen bonds between C=O bonds of the CDs and functional groups of the matrices, such as hydroxyl, carboxyl, and amine. The fixation effect allows the luminescent centers to tremendously reduce their nonradiative relaxation and to prolong the afterglow lifetimes of CDs. As an added benefit, the afterglow CD-doped polymer composites not only can be used for the preparation of films but also can be utilized for the creation of nanofibers. By taking advantage of electrospinning, He and coworkers reported the synthesis of CDs/PVA nanofibers with a diameter in the range of 20-40 nm [63]. They found that the as-prepared nanofibers can retain the afterglow luminescence of the CDs, enabling their widespread applications in the field of optoelectronics. However, an intrinsic limitation of polymer encapsulation strategy is associated with the hygroscopic nature of the polymer matrices. This effect imparts the afterglow luminescence of the resulting CD/polymer composites with weak tolerance to humility in the air [68].

As inspired by the structure of polymers, small molecules that can form hydrogen bonding network with surface subfluorophores of CDs has a similar ability to protect their excited triplet states (Figure 3(b)). The formed cross-linked hydrogen bonding networks serve as a rigid framework to encapsulate the CDs to decrease the nonradiative deactivation of the excited triplet species, enabling the generation of afterglow luminescence from the resulting CDs@molecule networks. In 2016, a team led by Li et al. reported the generation of afterglow luminescence of N-doped CDs with an average lifetime up to 0.93 s by heating them with urea at 155°C for 6 h [43]. The mechanism investigation suggests that *in situ* produced biuret via heating of urea can form hydrogen bonding with C=N/C=O bonds on the surface of N-doped CDs, allowing the formation of highly cross-linked CD-biuret networks (Figure 3(c)). In addition, they found that the urea residues can further recrystallize on the surface of CD@biuret to provide an additional rigid matrix layer to suppress the nonradiative decay channels. Later, Lin and coworkers further extended this strategy and achieved full-color ultralong afterglow of CDs [44].

Similar results were reported by Gao et al. with the use of melamine to form three-dimensional networks to encapsulate N-doped CDs to reduce the nonradiative deactivation of the excited triplet excitons (Figure 3(d)) [49]. The authors claimed that the fixation effect can promote the average lifetime and quantum yield of the afterglow luminescence of the N-doped CDs from 269 to 664 ms and from 20% to 25%, respectively. As an added merit, the afterglow luminescence can exist in the presence of water. A more interesting work that water can activate the afterglow luminescence of CDs in the presence of cyanuric acid (CA) was reported by Li and coworkers [29]. The authors elucidated that a layer of highly ordered water (nonfreezing bound water) can form between cyanuric acid particles and CDs to act as a bridge to effectively rigidify the two components (Figures 3(e) and 3(f)). This strong interaction leads to the formation of rigidified luminescent subunits of CDs, thereby giving rise to the enhanced afterglow luminescence in the presence of limited water. However, once there is presence of bulk water in the CD/CA composites, the afterglow luminescence will be quenched. In 2018, a similar work was reported by Tan and coworkers, who observed that CD/CA composites show pH-dependent afterglow. This observation is ascribed to the fact that CAs are prone to be neutralized by NaOH, and the resultant cyanurate ions tend to form stronger hydrogen-bonded networks to further rigidify the excited triplet excitons, leading to enhanced afterglow intensity [69].

#### 3.1.2. Complexing Interaction Activation

In addition to polymers and small organic molecules, inorganic salts are also useful as a matrix for activating the afterglow luminescence of CDs. In this case, the inorganic salts not only can form a protective layer to separate the excited triplet species from the environmental quenchers, such as O_2_ and humanity, but also can form complexing interactions with the functional groups on the surface of CDs via the constituent metal ions (M^n+^). This joint effect provides strong protection of the excited triplet excitons of CDs from nonradiative deactivation [70]. The perturbation of M^n+^ ions on the surface subfluorophores has a remarkable influence on the afterglow luminescence of the embedded CDs due to its impact on the spin-orbit coupling [71]. Dong et al. have pioneered the application of inorganic salts to activate the afterglow luminescence of CDs [27]. In their work, they found that the dispersion of CDs into the host material of KAl(SO_4_)_2_·x(H_2_O) can lead to an average afterglow lifetime of 655 ms for the CDs. The mechanistic study suggested that both the KAl(SO_4_)_2_ and crystal water molecules make contributions to rigidify the luminescent units of the CDs. Thereafter, a diversity of inorganic salts such as NaCl has shown the ability to activate the afterglow of CDs [72].

An intriguing work was recently reported by Green et al., who systematically demonstrated the dependence of the afterglow intensity and lifetime of embedded CDs on the mass of the metal cation and size of the anions of inorganic hosts [73]. By making use of alkaline earth carbonates, sulfates, and oxalates as host matrices, they found that the relative ratio of phosphorescence to fluorescence was increased with the cation atomic number. Meanwhile, the increase in the relative intensity of phosphorescence to fluorescence is accompanied by a decrease in the lifetime of the phosphorescence. They attribute these findings to the fact that heavier metal atoms can trigger higher rate constants for both ISC and radiative relaxation from $T_{1}$ to $S_{0}$ [74]. Besides, they showed that for the same cation, the size of anions has a negative influence on its ability to activate phosphoresce due to the increased distance between the perturbing nucleus and the electron that undergoes spin inversion. These findings suggest the advantages of the utilization of inorganic salts to controllably activate the afterglow luminescence of CDs via the choice of appropriate cations and anions. However, the instability of the inorganic matrices in the presence of water affects the stability of the optical attributes of CDs, indicative of the drawbacks of this strategy. Additional treatment of the CDs@inorganic salts to enable the growth of a water-tolerant passivation layer may provide a much-needed solution to this challenging issue.

#### 3.1.3. Covalent Bonding Activation

**Figure 4:**
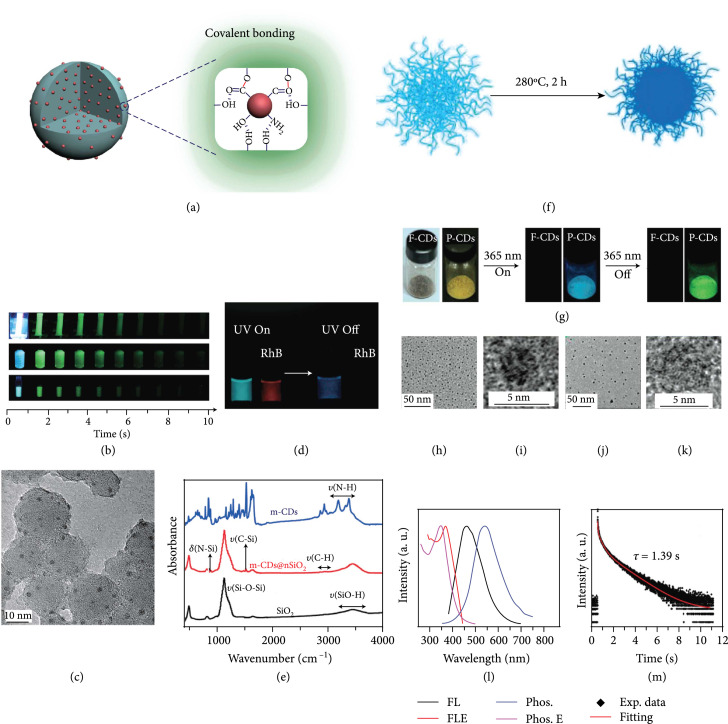
(a) Schematic representation of the activation of the afterglow luminescence of CDs via covalent fixation effect. (b) Photographs of CDs@SiO_2_ mixture in dialysis against deionized water (upper row), an aqueous dispersion of CDs@SiO_2_ (middle row), and CDs@SiO_2_ powder (bottom row) with turn-on and turn-off of UV excitation at different delay times (adapted and copyright permission [46], American Chemical Society). (c) TEM image of nSiO_2_ covalently modified with CDs. (d) Photographs of the dispersion of CDs@nSiO_2_ and RhB solution with and intermediate removal of UV excitation (365 nm). (e) FT-IR profile of CDs, nSiO_2_, and CDs@nSiO_2_ (adapted and copyright permission [75] (c–e), American Chemical Society). (f) Schematic representation of the conversion of fluorescent CDs (F-CDs) to phosphorescent CDs (P-CDs) via high-temperature heating. (g) Photographs of P-CDs upon turn-on and turn-off of UV excitation. (h, i) TEM and HRTEM images of F-CDs. (j, k) TEM and HRTEM of P-CDs. (l) Normalized fluorescence (black curve) and phosphorescence (blue curve) profiles of P-CD powder and corresponding excitation spectra (red line for fluorescence at 461 nm, pink line for phosphorescence at 538 nm) (adapted and copyright permission [76] (f–m), Wiley-VCH Verlag GmbH & Co. KGaA).

In comparison to hydrogen bonding and complexing interactions, covalent bonding is expected to have extraordinary ability to fix the triplet excitons due to its strong bonding strength, thereby giving rise to remarkable activation potency for the afterglow luminescence of CDs (Figure 4(a)). The formation of covalent bonding between matrices and CDs can initialize with the formation of hydrogen bonding interactions followed by conversion of the hydrogen bonds into covalent bonding at high temperatures or with prolonging reaction time. Typical examples can be found in the preparation of CDs@SiO_2_ nanocomposites which are often synthesized on the basis of hydrolysis and condensation of tetraethyl orthosilicate in the presence of CDs in an alkaline aqueous solution. The functional groups on the surface of CDs, such as –OH and –NH_2_, can directly participate in the condensation reaction, serving as nucleation sites for the growth of the SiO_2_ matrix. This accounts for the observation of characteristic vibrations of Si–O–C bonds at 1260 and 931 cm^-1^ in the Fourier transform infrared (FT-IR) spectroscopy of CDs@SiO_2_ [42]. Besides, the formation of Si–O–C bonds at room temperature can also be supported by X-ray photoelectron spectroscopy in which a band at 103.0 eV in the Si 2p spectrum can be observed. The formation of Si–O–C bonds in the synthesis of CDs@SiO_2_ nanocomposite was found to be associated with the synthetic temperatures. High temperatures seem to be useful for further promotion of the dehydration reaction between CDs and SiO_2_ matrix. In 2019, Li et al. developed an interesting variation of the conventional Stöber method that enables the formation of CDs@SiO_2_ at 100°C [46]. They reported that the afterglow luminescence of the as-prepared CDs@SiO_2_ nanocomposites shows a record-long lifetime of 1.64 s in an aqueous solution and displays considerable tolerance toward O_2_ and transition M^n+^ (Figure 4(b)). This work also suggests that the improved covalent bonding not only shows the ability to stabilize the excited triplet species in CDs but also shows action on improving both ISC and RISC by decreasing the energy gap between $S_{1}$ and $T_{1}$.

Covalent fixation of CDs on the surface of SiO_2_ nanoparticles has also been utilized to activate RT phosphorescence of CDs. In 2018, Jiang and coworkers have reported the use of the hydrothermal reaction to covalently immobilize CDs on the surface of colloidal nanosilica (nSiO_2_) and observed a long lifetime of the afterglow emission up to 0.703 s in a water dispersion [75]. In addition to Si–O–C bonds, this work suggests that the covalent bonding between m-CDs and nSiO_2_ can take place via the formation of C–Si and N–Si bonds under hydrothermal conditions. The strong covalent fixation provides direct evidence for effective stabilization of the excited triplet species on the surface of CDs, thereby giving rise to improved triplet relevant emissions. On a separate note, the strong covalent stabilization strength endows the afterglow emission with a TADF mechanism other than RTP that was often observed with the use of hydrogen bonding as the activation force alone.

Stronger activation potency of covalent bonding than hydrogen bonding in the generation of afterglow luminescence of CDs was also observed in CD-doped polymer composites. In 2018, Tian and coworkers have reported that thermal annealing of CDs in PVA matrix at 200°C can promote the formation of chemical bonding between the two components [77]. The chemical bonding helps stabilize the triplet emissive species, enabling longer afterglow luminescence in comparison to the one which was prepared under the same conditions but subsequently treated at a low temperature of 80°C.

Alternatively, covalent bonding can be formed between CDs and matrixes derived from small molecules to improve the afterglow luminescence of the CD component. In 2018, Li et al. have demonstrated that the treatment of a mixture of heteroatom-free CDs and boric acid (BA) at 180°C for 5 h led to the formation of amorphous glassy composites of CDs/BA with a long phosphorescence lifetime up to 2.26 s [39]. In addition, the phosphorescence quantum yield was found as high as 17.5%. They attributed the improved afterglow performance to the formation of boron–carbon bonds due to the observation of a new vibration band at 948 cm^-1^ in the FT-IR profile and a signal at 192.6 eV in the XPS spectrum. Later, this strategy was extended to the synthesis of CDs@B_2_O_3_ nanocomposites by Xu and coworkers, who observed a TADF afterglow luminescence at 480 nm with a lifetime of 805.94 ms at 273 K [78]. Similarly, the main limitation of the strategy lies in the fact that the host matrix of B_2_O_3_ is unstable in the presence of water, enabling the afterglow to be quenchable by moisture in the practical applications.

Inspired by the results of polymer-matrix-activated afterglow luminescence of CDs, exquisite structure modulation of presynthesized CDs via high-temperature heating may trigger their conversion from fluorescent CDs to phosphorescent CDs. Such heating treatment is likely to provide a balance between carbonization of the graphitized core and formation of improved cross-linked surface intertwined polymers. Both factors show the promise to stabilize the excited triplet species in CDs. Notably, the formation of a highly cross-linked surface layer can also prevent the occurrence of aggregation-induced fluorescence quenching. In 2018, Jiang et al. reported that heating treatment (280°C for 2 h) of fluorescent CDs leads to the emergence of RTP (Figures 4(f) and 4(g)) [76]. The change in the physical attributes of the treated CDs can be inferred from the increase in their average size from 3.2 to 6.4 nm (Figures 4(h)–4(k)). Spectroscopic analysis suggests an average lifetime of 1.39 s of the emergent RTP at 538 nm in the form of powder (Figures 4(l) and 4(m)).

### 3.2. One-Step Activation Method

Instead of postsynthetic treatment of CDs, one-step methods have also been explored to activate afterglow CDs with or without the use of extraprotective matrices. In one-step activation methods, the protective matrices can be the synthetic medium or side products simultaneously formed with the generation of emissive CDs at high temperatures. Similar to that in two-step activation methods, the protective matrices were found to have the utility of stabilization of the excited triplet species of CDs, rendering the resulting CDs@matrix nanocomposites with afterglow luminescence. In comparison to two-step activation, one-step activation can afford largely simplified synthetic protocols for the preparation of afterglow CDs@matrix. According to the nature of the matrices, they can generally be divided into four classes, including molten salt activation, layered inorganic compound activation, zeolite activation, and polymer matrix activation.

#### 3.2.1. Molten Salt Encapsulation Activation

**Figure 5:**
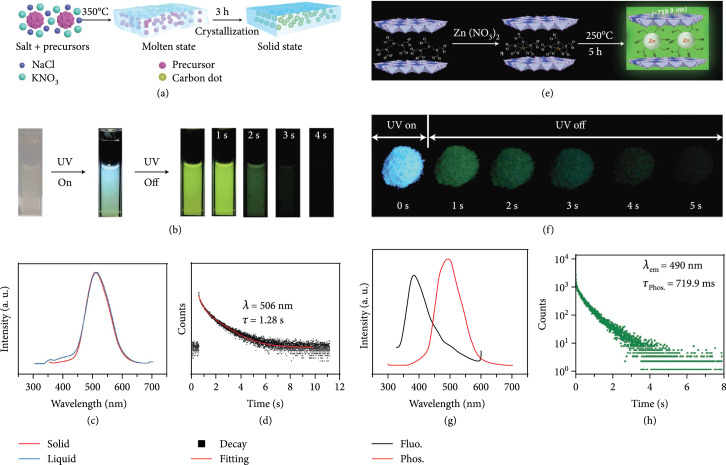
(a) Schematic representation of activation of the afterglow luminescence of CDs via MS encapsulation. In this strategy, a mixture of molten salts and CD precursors was heated at high temperatures for several to enable the carbonization of the precursors. When cooling to room temperature, molten salts were capped on the surface of the resulting CDs, allowing the formation of CDs@MS composites (adapted and copyright permission [80], Royal Society of Chemistry). (b) Photographs of CDs@MP dispersion under daylight, UV light (365 nm) on, and UV light off with different delay times, respectively. (c) Phosphorescence profile comparison of the CDs@MP in the solid and solution states. (d) The decay curve of the phosphorescence of the CDs@MP at 506 nm (adapted and copyright permission [52] (b–d), American Chemical Society). (e) Schematic representation of activation of the afterglow luminescence of CDs via host-guest encapsulation. In this strategy, CD precursors (guest molecules) were first inserted into the nanospace of layered compounds (host matrices) followed by heating treatment at high temperatures. In the heating treatment, the precursors were carbonized into CDs which are simultaneously encapsulated by the host inorganic matrices. (b) Photographs of CDs@LDHs under UV excitation and ceasing the excitation as a function of time. (c) The comparison of fluorescence and phosphorescence profiles of the resulting CDs@LDHs. (d) Phosphorescence decay behavior of the CDs@LDHs at 490 nm (adapted and copyright permission [81] (e–h), Royal Society of Chemistry).

Molten salt (MS) is a class of inorganic compounds that undergo a phase transformation from solid under ambient conditions to liquid at high temperatures. The melting temperature of molten salts can be precisely controlled through the choice of a combination of readily available salts, allowing carbonization temperatures of precursors to be facilely controlled. The use of molten salt as a reaction medium has proven effective in the synthesis of fluorescent CDs [79]. In 2019, Wang et al.’s group has extended this strategy to the preparation of afterglow CDs@MS nanocomposite via enhancing the reaction temperature to 350°C (Figure 5(a)) [80]. In this study, they demonstrated that the CDs produced via the carbonization of small organic molecules at the high temperature can be subsequently encapsulated by the solid molten salts at room temperature. The crystalline lattices of MS activated the afterglow luminescence of the CDs via a combination of effective locking of the triplet excitons and separation of the surface subfluorophores from surrounding quenchers. When compared with previous work on MS-assisted synthesis of fluorescent CDs, one can get the conclusion that reaction-temperature-controlled surface oxidation modulation plays a vital role in determining the optical attributes of CDs. Despite the attractiveness, the use of MS in the preparation of CD-based afterglow nanocomposites is hindered by the hygroscopic nature of the used MS, which enables the weak photoluminescence stability of the resultant CDs@MS nanocomposites. This concern can be partially addressed by doping MgCl_2_ and KH_2_PO_4_ into the MS in the carbonization synthesis of CDs, as supported by their follow-up work [52]. Owing to the poor solubility of Mg-PO_4_ salts in water, the tolerance of the afterglow luminescence toward water has been considerably improved, even leading to the observation of a bright long-lived yellow RTP in an aqueous solution of the resulting CD@MP after ceasing the UV excitation (Figures 5(b)–5(d)). As inspired by these findings, emissive transition M^n+^, such as Mn^2+^, may be possibly added into the MS to trigger energy transfer from the phosphorescence of CDs to the doped M^n+^ to realize afterglow emission modulation.

#### 3.2.2. Host-Guest Encapsulation Activation

**Figure 6:**
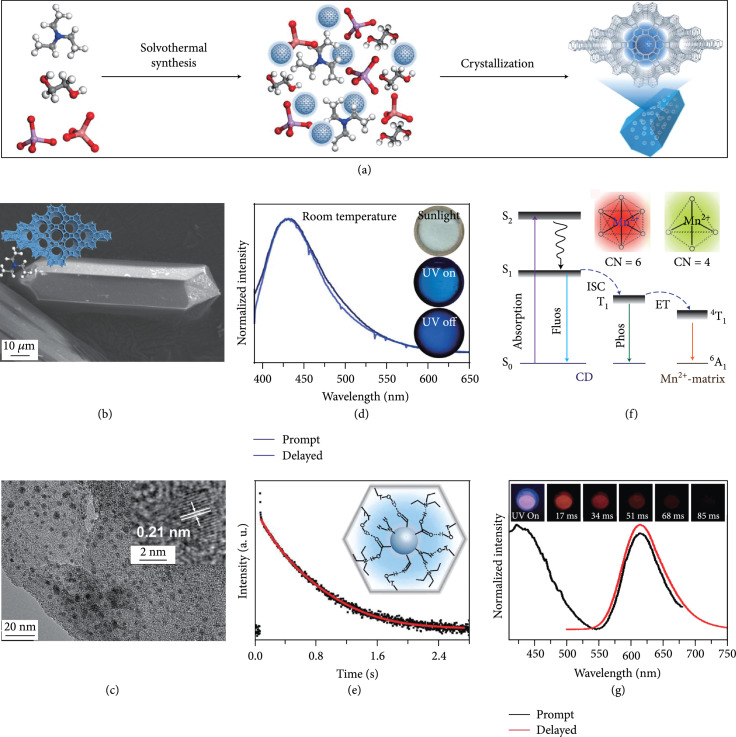
(a) Schematic representation of activation of the afterglow luminescence of CDs via zeolite encapsulation. In this strategy, organic species in the synthesis of zeolite, such as solvent and structure-directing agent, were first carbonized into CDs which were then encapsulated with the *in situ* formed zeolite matrices at high temperatures. (b, c) SEM and TEM images of CDs@AlPO-5 composites. Inset in (b): the structures of the inorganic framework and SDA. Inset in (c): HRTEM of a selected CD showing crystal lattices. (d) The profile comparison of prompt (deep blue line) and delayed photoluminescence (blue line) of CDs@AlPO-5 under excitation at 370 nm. Inset: photographs of CDs@AlPO-5 under daylight, UV on, and UV off states. (e) The decay curve of the DF of CDs@AlPO-5 at 430 nm. Inset: schematic illustration of the interaction between the terminal –OH groups of the interrupted zeolite framework and the functional groups on the surface of the CDs (adapted and copyright permission [28] (a–e), American Association for Advancement of Science). (f) Schematic energy level diagrams presenting the energy transfer of the phosphorescence of CDs to the Mn^2+^ ions in the zeolite matrix of MnAPO-CJ50. Inset: the dependence of the luminescence of Mn^2+^ ions on their coordination configuration. (f) Prompt (black line) and delayed (red line) photoluminescence profiles of the CDs@MnAPO-CJ50 under excitation at 360 nm. Inset: photographs of the CDs@MnAPO-CJ50 under UV excitation and ceasing the excitation with different delay time (adapted and copyright permission [82] (f, g), Wiley-VCH Verlag GmbH & Co. KGaA).

The host-guest encapsulation method is another alternative technique for activation of the afterglow luminescence of CDs in the form of nanocomposites. It usually involves two steps: insertion of CD precursors into the nanospace of host matrices and calcination of the resulting host-guest compounds at high temperatures (Figure 6(e)). In 2017, Bai et al. first reported the use of layered double hydroxides (LDHs) as host matrices for inserting EDTA·2Na by taking advantage of their layered structures [83]. Calcination of the resulting EDTA-LDHs at 300°C for 4 h led to the formation of CDs@LDHs with a long-lived emission at 525 nm (386.8 ms). They ascribed the generation of the strong afterglow luminescence to a protective effect provided by the confined and rigid interlayered nanospace. In addition, the strong complexing interaction between the host cations and the surface subfluorophores may promise to increase the afterglow emission intensity by elevating the ISC probability. This effect was later verified by their follow-up work in which EDTA·Zn was used to replace EDTA as the precursor for CD synthesis [81]. They reported that the lifetime of afterglow luminescence of the as-prepared Zn-CDs@LDHs was estimated to be 719.9 ms (Figures 6(f)–6(h)), approximately two times longer than that of the CDs@LDHs. In addition to LDHs, nanoclays were also reported as a host lattice for the activation of afterglow luminescence of CDs due to the fact that their layered structures have a similar function in the accommodation of CD precursors and immobilization of the excited triplet excitons of the resulting CDs [84].

#### 3.2.3. Zeolite Encapsulation Activation

Zeolites are microporous crystalline solids of aluminosilicates or aluminophosphates with well-defined structures and uniformed pores. They are attractive as host matrices for encapsulation of CDs and activation of their afterglow luminescence [85, 86]. Recently, Liu et al.’s group led the pioneering effort on developing a one-step hydrothermal/solvothermal encapsulation method for the synthesis of afterglow CDs@zeolite composites [28]. In this strategy, the hydrothermal/solvothermal conditions first enable the carbonization of the organic species into CDs that are subsequently encapsulated by zeolite matrices formed in the synthetic mixture (Figure 6(a)). The organic species include organic structure-directing agents (such as triethylamine) and the solvents (such as triethylene glycol). A combination of SEM and TEM characterization suggests that CDs with high crystallinity were encapsulated in the zeolite of AlPO-5 (Figures 6(b) and 6(c)). The CDs@zeolite composites showed ultralong blue TADF with a lifetime up to 350 ms as a result of effective suppression of the nonradiative decay process (Figures 6(d) and 6(e)). The remarkable stabilization ability of AlPO-5 toward excited triplet excitons is possibly due to the formation of hydrogen and covalent bonding between the terminal –OH groups in the micropore and the surface functional groups of the CDs (inset, Figure 6(e)).

This method shows considerable flexibility in tuning the properties of CDs@zeolite composites [87]. First, the afterglow quantum yield and lifetime can be modulated via the choice of the organic species in zeolite synthesis [88]. Moreover, there are multiple choices of the zeolite matrices with tunable terminal groups for encapsulating the *in situ* formed CDs, imparting the feasibility in control of the interaction strength between zeolite and CDs to tune the afterglow attribute of the nanocomposites [89]. As an added benefit, Mn^2+^ ions can be doped into the host zeolite matrices to lead to a shift of the afterglow emission from blue to red benefiting from energy transfer from the CDs to the doped Mn^2+^ ions (Figure 6(f)). Notably, the afterglow emission for Mn^2+^ can be further tuned from 620 to 530 nm by changing the coordination geometry of the doped Mn^2+^ ions from octahedral to tetrahedral through the choice of appropriate host matrices (inset, Figure 6(f)) [82].

In addition, the loading efficiency of CDs in the host zeolite matrices was demonstrated to be enhanced by a solvent-free thermal crystallization strategy. In a recent report, Zhang and coworkers reported that solvent-free thermal crystallization of solid raw materials of zeolite and precursor CDs at 220°C for 20 h led to a loading capacity of CDs up to 1.7 wt% [90]. In the synthesis, they found a gradual increase in the afterglow emission of CDs with prolonging the reaction time as an outcome of enhanced fixation interaction between the CDs and the host zeolite matrix.

### 3.3. Self-Protective Activation

**Figure 7:**
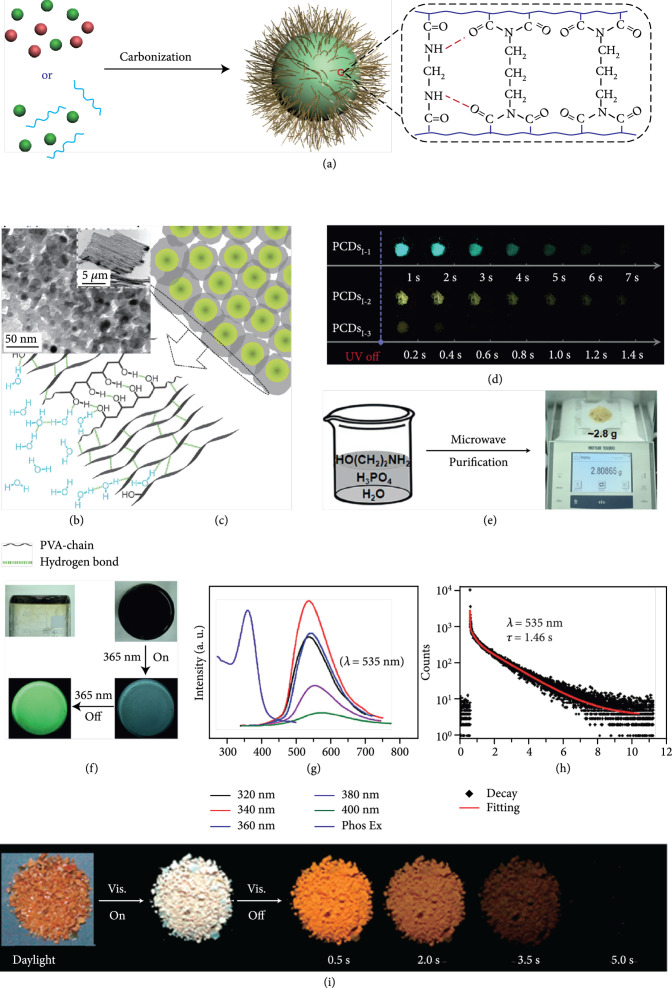
(a) Schematic representation of the synthesis of self-protective afterglow CDs. In this strategy, a mixture of molecules or molecules and polymers was heated at high temperatures for carbonization synthesis of afterglow CDs. The unique structural characteristic of the resulting CDs is the presence of a layer of highly cross-linked polymer-like chains on the surface of the graphitized core. The surface layer can serve as a protective matrix to stabilize excited triplet species of CDs due to the existence of covalent and hydrogen bonding between the polymer-like chains. (b) TEM image of self-protective afterglow CDs after being dispersed in ethanol. Note that the CDs were prepared by the carbonization of PVA and ethylene diamine under hydrothermal conditions (200°C, 8 h). Inset: sheet-like CD aggregates. (c) Schematic illustration of a stabilization effect of the excited triplet states by the surface polymer-like chains (adapted and copyright permission [91] (b, c), Royal Society of Chemistry). (d) Photographs of self-protective afterglow CDs at different delay times after the removal of UV excitation. Note that PCD_i-1_, PCD_i-2_, and PCD_i-3_ were prepared with the use of mixtures of PAA and EDA, PAA and EAM, and PAA and ethylene glycol as precursors, respectively (adapted and copyright permission [45], Wiley-VCH Verlag GmbH & Co. KGaA). (e) Schematic representation of the preparation of self-protective afterglow CDs via a microwave-assisted heating method. (f) Photographs of self-protective afterglow CDs under daylight, UV excitation on, and UV excitation off. (g) Phosphorescence profiles of the CD powder as a function of excitation wavelengths and the excitation profile at the phosphorescence emission of 535 nm. (h) The decay curve of the phosphorescence of CDs at 535 nm (adapted and copyright permission [30] (e–h), Wiley-VCH Verlag GmbH & Co. KGaA). (i) Photographs of self-protective afterglow CDs under daylight, UV excitation on, and UV excitation off at different delay times. Note that the afterglow CDs were prepared by microwave irradiation of a mixture of L-aspartic acid (AA) in the presence of ammonia (adapted and copyright permission [92], MDPI).

Over the past few years, substantial research efforts have been devoted to exploring synthetic strategies toward self-protective afterglow CDs with tunable color output (Figure 7(a)). The synthesis of self-protective afterglow CDs needs addressing at least two challenging problems. One is the self-quenching of the luminescence of CDs in the solid-state, and the other is the deactivation of the excited triplet species by nonradiative decay processes in the absence of additional protective matrices.

Owing to the important role of surface intertwined polymer chains in the stabilization of the excited triplet species, polymers can be directly used as a component of precursor in afterglow CD synthesis as a result of the consideration that incompletely carbonized polymer chains may be presented on the surface of the resulting CDs. In 2017, Chen et al. first demonstrated the use of polyvinyl alcohol (PVA) for the synthesis of afterglow CDs in the presence of EDA via a hydrothermal reaction (220°C, 10 h) [91]. Their results showed that PVA-chains were presented at the surface of the as-prepared CDs, which not only can activate the afterglow luminescence of the CDs but also can impede penetration of moisture and oxygen from the surface to the embedded emissive species through the formation of hydrogen bonding (Figures 7(b) and 7(c)). This structure feature endows the self-protective CDs with RTP at 564 nm in the aggregation state with an average lifetime of 13.4 ms.

Later, Tao et al.’s group reported the use of PAA and EDA as precursors to afford afterglow CDs under hydrothermal conditions (200°C, 8 h) (Figure 7(d)) [45]. Their theoretical and experimental results suggested that covalently cross-linked frameworks formed via the hydrothermal reaction of the two components can improve the ISC for effectively populating triplet excitons as well as enhance their stability. Similar results were lately reported by Zhu et al., who realized the synthesis of RTP CDs via the use of a mixture of polyvinylpyrrolidone and urea as shell precursor in the presence of CD cores [93]. Interestingly, the use of polymers in CD synthesis not only shows the possibility of activation of the phosphorescence but also displays the promise to tune the fluorescence attributes, including quantum yield and emission wavelength [94]. Alternatively, small organic molecules also show great promise as precursors for the synthesis of afterglow CDs via exquisite control of synthetic conditions. Among the synthetic parameters, carbonization temperatures are found to be crucial for the generation of self-protective afterglow CDs. The combination of microwave-assisted heating and the choice of reaction medium has proven effective in the facile and large-scale production of afterglow CDs. In 2018, Jiang and coworkers reported microwave-assisted synthesis (750 W, 5 min) of afterglow CDs via the use of a mixture of EAM and phosphoric acid as the precursors (Figure 7(e)) [30]. They found that the as-prepared CDs showed a strong green afterglow luminescence at 535 nm with a lifetime up to 1.46 s in the solid-state (Figures 7(f)–7(h)). In this synthesis, phosphoric acid was found to be important not only in the achievement of P doping but also in controlling reaction temperature. This method was later extended by Yang and coworkers, who realized the synthesis of afterglow CDs by replacing EAM with other N-containing precursors, such as triethanolamine, while keeping other conditions almost the same [51, 95]. Alternatively, phosphoric acid can also be replaced by other phosphate-containing compounds, such as phytic acid. For example, Qi et al. recently showed that microwave-assisted heating (800 W, 2 min) of a mixture of phytic acid and triethylenetetramine led to the production of self-protective afterglow CDs as well, exhibiting a maximum emission band at 535 nm with a long average lifetime up to 750 ms [96]. In a recent work by Hu et al. [92], microwave-assisted heating of L-aspartic acid in the presence of ammonia was elucidated to be useful for preparing CDs with a unique orange afterglow luminescence (585 nm) with an average lifetime of 240.8 ms under excitation at 420 nm (Figure 7(i)). This finding suggests that the color output of the afterglow luminescence of CDs is likely to be manipulated by proper selection of the combination of CD precursors.

As a separate note, the carbonization of small molecule precursors under hydrothermal or normal conditions in some cases can also be utilized for the synthesis of self-protective afterglow CDs. However, due to the relatively low reaction temperatures in such conditions (usually <220°C), long reaction times are necessary to result in the formation of a compact core surrounding by highly cross-linked polymer chains. In 2019, Gao et al.’s group has demonstrated that hydrothermal treatment of EDTA as a single precursor at 200°C for 5 h led to the formation of self-protective CDs with a maximum phosphorescence emission at 540 nm under excitation at 364 nm [98]. The emission was found to show a triexponential decay behavior with an average lifetime of 1.51 s. Obviously, the multiple-channel decay behavior suggests that the afterglow luminescence may originate from different emissive species. In a recent follow-up work [99], they presented that direct heating of an alkaline aqueous mixture of glucose and L-aspartic acid until the formation of a pale-yellow solid can also afford self-protective afterglow CDs. These CDs display a broadband emission at 515 nm with a short average lifetime of 747 ms upon excitation at 320 nm. The decrease in the lifetime of the afterglow luminescence was possibly due to the formation of loose core and poorly cross-linked surface polymer-like chains as a result of the low reaction temperature.

Owing to the presence of abundant surface hydrophilic functional groups, self-protective afterglow CDs often show hygroscopic nature and exhibit the inability to emit afterglow luminescence in the presence of moisture. In addition, the hydrophilic functional groups show the promise to complex with M^n+^, leading to a quenching effect on the photoluminescence of CDs. These challenges need to be solved prior to the realization of their utility in practical applications. One possible solution is to perform secondary protection with the use of additional matrices, such as SiO_2_, as supported by the discussion presented in the section of covalent bonding activation of the afterglow luminescence of CDs.

## 4. Emerging Afterglow Luminescence Properties

Carbonization of molecule or polymer precursors at high temperatures leads to the formation of different kinds of subfluorophores on the surface of CDs. For example, a variety of subfluorophores, including C=N, N=O, –NH_2_, C–N, and C–N, may derive from N-containing organic molecules. Moreover, these functional groups in the confined nanospace may further interact with each other through electron overlap to create additional $T_{1}$ energy levels. A combination of the presence of abundant $T_{1}$ energy levels, binary afterglow mechanism, and the susceptibility of the excited triplet states to external stimuli allows CDs to exhibit intriguing afterglow luminescence.

### 4.1. Excitation-Dependent Afterglow Luminescence

The ability to manipulate afterglow color output of optical materials is important for their applications in biological labeling, security systems, and optoelectronic devices. Due to the lack of abundant tunable structures, afterglow luminescence of inorganic materials is often difficult to be adjusted with a fixed doping combination [100, 101]. Construction of multiple-component platforms, for example, encapsulation of multiple emissive dye molecules into long-lived luminescent MOFs, has shown promise as a tool to modulate their afterglow luminescence [102].

**Figure 8:**
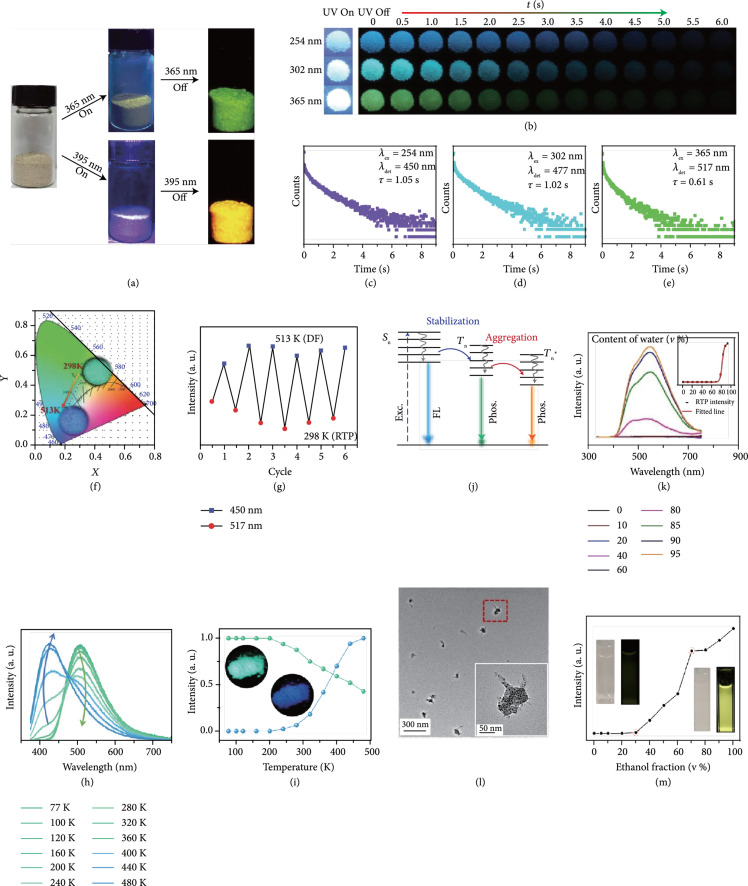
(a) Photographs of CDs@molten salt upon turn-on and turn-off of the excitation at wavelengths of 356 and 395 nm, respectively (adapted and copyright permission [80], Royal Society of Chemistry). (b) Photographs of CDs@nanoclay under excitation on and off at wavelengths of 254, 302, and 365 nm, respectively (c) Afterglow decay profiles of CDs@clay at emission wavelengths of 450, 477, and 517 nm. (f) CIE coordinate diagram showing the change in afterglow color output of CDs@clay upon changing temperature from 298 to 513 K (under excitation at 365 nm). (g) Reversibility of afterglow transition from cyan (at 517 nm) to blue (at 450 nm) upon alternative change of temperature in the window (adapted and copyright permission [84] (b–g), Royal Society of Chemistry). (h) Afterglow luminescence profiles of CDs@SiO_2_ as a function of measurement temperatures (excitation at 365 nm). (i) Normalized RTP and TADF emission intensity as a function of temperature (excitation at 365 nm). Inset: typical afterglow color output originating from RTP (green) and TADF (blue) (adapted and copyright permission [97] (h, i), Royal Society of Chemistry). (j) Schematic energy level diagrams showing the generation of a new triplet state ($T^{*}$) as a result of the aggregation of CDs. (k) RTP emission profiles of a THF dispersion of TA-CDs with the addition of different volumes of water. Inset: the dependence of RTP intensity on the added water content (v%) in the CD mixture. (c) TEM image of CDs in a mixture of THF:H_2_O (1 : 9 v%). Inset: enlarged TEM of an aggregate comprising CDs (adapted and copyright permission [40] (j–l), Wiley-VCH Verlag GmbH & Co. KGaA). (m) Phosphorescence profiles of CDs@MP in a mixture of HCl/ethanol with different contents of ethanol (v%). Insets: photographs of CDs@MP mixtures under daylight and UV light (365 nm) (adapted and copyright permission [52], American Chemical Society).

Owing to their excitation-dependent RTP nature, CDs show tunable afterglow luminescence without the need for changing their intrinsic physical parameters, such as composition and oxidization degree. The origin of the excitation-dependent RTP is likely due to the presence of multiple triplet excited states in CDs. As demonstrated by Wang and coworkers in 2019, the afterglow luminescence of N-doped CDs@MS composites gradually shifts from 510 to 573 nm by adjusting the excitation wavelength from 360 to 440 nm. More interestingly, green and orange afterglow luminescence was observed from the same CDs@MS upon the excitation at wavelengths of 365 and 395 nm, respectively (Figure 8(a)) [80]. This mechanism can also be used to explain the excitation-dependent RTP of self-protective N-doped and N,P-codoped CDs [76, 92].

Another plausible explanation is due to the simultaneous existence of phosphorescence and DF which have different optimal excitation wavelengths. For example, Lin et al. recently reported that N-doped CD-biuret@urea composites show blue DF centered at 430 nm and green phosphorescence centered at 500 upon excitation at 254 and 365 nm, respectively [44]. In a parallel development, Deng et al. have reported the excitation-dependent afterglow luminescence of CDs confined in nanoclays in 2019 [84]. In their study, they found that the afterglow luminescence gradually shifts from 450 (blue) to 530 nm (green) when changing the excitation from 254 to 380 nm (Figures 8(b)–8(e)). According to the difference in the energy gap ($\Delta E_{\text{ST}}$) between the singlet ($S_{1}$) and triplet ($T_{1}$) states, TADF accounts for the afterglow luminescence when using the excitation wavelength in the region of 254 to 302 nm due to the presence of small $\Delta E_{\text{ST}}$ (<0.3 eV), while RTP dominates the afterglow luminescence upon excitation from 302 to 380 nm as a result of a large $\Delta E_{\text{ST}}$ (>0.3 eV).

### 4.2. Temperature-Dependent Afterglow Luminescence

As was already mentioned above, afterglow luminescence of CDs sometimes originates as a mixture of TADF and RTP under excitation. The temperature has an opposite impact on these two components. In addition, RTP has a longer emission wavelength in comparison to TADF. As a result, increasing the portion of TADF in the afterglow luminescence by elevating temperature can lead to a gradual blueshift in the afterglow luminescence profile. More importantly, a reverse transition can be achieved upon decreasing the temperature. In 2019, Deng et al. have demonstrated reversible afterglow luminescence of CDs@nanoclay composites from cyan (513 nm) to blue (450 nm) by changing the temperature in the range of 298 to 513 K (Figure 8(f)) [84]. The high reversibility is an outcome of the intact of the intrinsic attributes of the CDs during the experiment (Figure 8(g)). Similar results were observed from CDs@SiO_2_ nanocomposites by Sun and coworkers in 2020 (Figures 8(h) and 8(i)) [97]. Though the temperature-responsive afterglow luminescence of CDs can add considerable versatility in their applications, rational synthesis of CDs with a controlled combination of RTP and TADF is still a formidable challenge. To achieve this goal, the delicate modulation of the interaction between CDs and the protective matrices to balance the contribution of RTP and TADF in the afterglow luminescence is highly desirable in the preparation of CDs.

### 4.3. Aggregation-Induced Afterglow Luminescence

Aggregation-induced emission (AIE) is an interesting photophysical phenomenon reported by Luo and coworkers in 2001, who have demonstrated that nonemissive luminogens become highly emissive once their aggregates are formed in the solution [103]. Without surface protection, CDs are prone to aggregate at high concentrations or in the solid state due to the presence of the conjugation system of the graphitized cores. For fluorescence, aggregation of CDs can largely decrease the distance between the fluorescent centers, leading to a redshift in the emission profile or even the quenching of the emission due to the nonradiative recombination of charge carriers [104, 105]. In contrast, recent studies suggest that aggregation of CDs resulting from their π–π stacking not only holds promise to stabilize the $T_{1}$ excited state but also possibly leads to the formation of an additional triplet excited state ($T_{1}*$) (Figure 8(j)).

In 2019, Jiang and coworkers first reported the aggregation-induced yellow RTP from CDs which were prepared via the hydrothermal treatment of trimellitic acid (TA) at 260°C for 12 h [40]. In their study, they have reported that blue fluorescence was only observed from a THF dispersion of the CDs, whereas yellow RTP appeared when the water content (v%) in the mixture was higher than 80% (Figure 8(k)). The proposed aggregation-induced RTP mechanism was supported by TEM imaging in which randomly oriented CD aggregates were observed (Figure 8(i)). Another example was reported by Wang et al. in 2020, who have found that enhancing ethanol fraction in an acidic CD dispersion higher than 30% can also trigger the appearance of yellow RTP [52]. In this case, enhancing the ethanol fraction in the mixture first led to the formation of CDs@MS nanocomposites which underwent further aggregation and gave rise to bright yellow RTP in the resulting solution (Figure 8(m)).

## 5. Applications

In addition to afterglow luminescence, afterglow CDs have an additional ability to emit short-lived fluorescence upon UV light excitation. The dual-mode emissive nature, together with their high tunability makes afterglow CDs outstanding from other luminescent materials. In this section, we mainly present the application of afterglow CDs in the fields of sensing, bioimaging, anticounterfeiting, and data encryption benefiting from their unique optical properties.

### 5.1. Sensing

**Figure 9:**
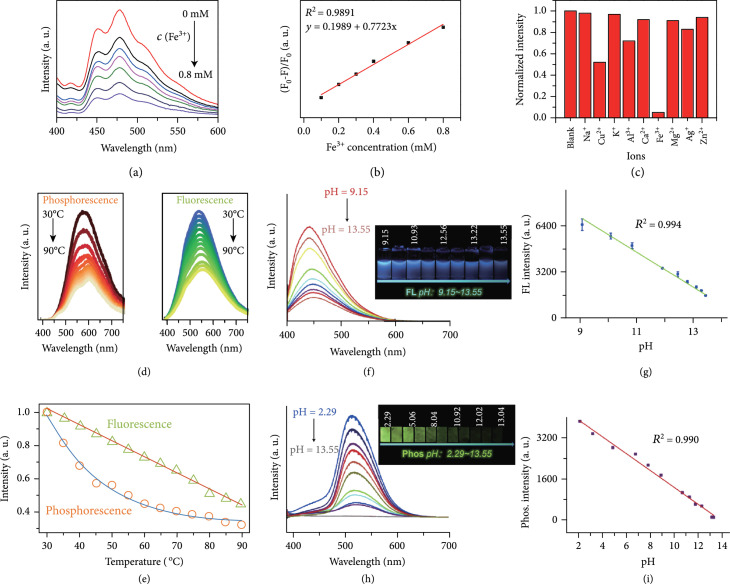
(a) Phosphorescence of a CD-CA dispersion as a function of the concentration of Fe^3+^ in the mixture (0, 0.1, 0.2, 0.3, 0.4, 0.5, and 0.8 mM). (b) Linear response of $\left(F_{0}-F\right)/F_{0}$ to the concentration of Fe^3+^ ions. (c) The selectivity of the measurement of Fe^3+^ on the basis of the quenching of the phosphorescence of CD-CA (adapted and copyright permission [29] (a–c), Nature Publishing Group). (d) The dual response of the fluorescence and phosphorescence of CD powder to the change of temperature in the range of 30-90°C. (e) The fitted curves of the fluorescence and phosphorescence intensities as a function of temperature (adapted and copyright permission [91] (d, e), Royal Society of Chemistry). (f, g) Measured and fitted fluorescence intensity of P-CDs to pH in the region of 9.15 to 13.55. Inset showing the fluorescence images of an aqueous P-CD dispersion at different pH values. (h, i) Measured and fitted phosphorescence intensity of P-CDs as a function of pH in the region of 2.29 to 13.55. Inset showing the phosphorescence images of P-CD coated papers at different pH values (adapted and copyright permission [51] (f–i), Elsevier B.V.).

In comparison with fluorescent CDs, afterglow CDs show considerable advantages in biosensing applications. First, taking advantage of the long-lived nature of the afterglow luminescence, the short-lived fluorescence, and scattering light allows to be easily separated from the profile via the use of a time-gated detection method. As a result, the detection limit promises to be significantly improved as a result of the absence of interference from background fluorescence. In 2018, Li and coworkers developed a solution method to detect Fe^3+^ ions based on their quenching ability toward the phosphorescence of CD@CA by the formation of nonfluorescent Fe^3+^-CD complexes (Figure 9(a)) [29]. Their results suggested that the quenching degrees of the phosphorescence of CD@CA show a linear response to the concentration of Fe^3+^ ions and selectively limits to the presence of Fe^3+^ ions in the analytic system (Figures 9(b) and 9(c)). Their findings also showed the inability of the fluorescence of CD@CA to quantitatively probe Fe^3+^ ions in a complicated mixture containing amino acids and proteins which can result in strong background fluorescence. On a separate note, the phosphorescence of CD-Fe^3+^ complexes can be recovered by the addition of phosphate-containing compounds into the mixture to strip the quenchers of Fe^3+^ ions. On the basis of this mechanism, Yan and coworkers have developed a phosphorescence “off-to-on” strategy to realize the detection of adenosine-5'-triphosphate with a detection limit of down to 14 *μ*M [106]. Similar “off-to-on” designs of the phosphorescence of CDs have been extended to highly selective detection of other species in an aqueous solution, including alpha fetal protein, Hg^2+^, and target ssDNA. In the analysis, 5-fluorouracil-labeled ssDNA and graphene oxide sheets were often used as quenchers [107, 108].

Notably, once both the fluorescence and phosphorescence of CDs give rise to changes in response to the alteration of stimuli or analytes, quantitative sensing holds promise to be simultaneously carried out from dual channels. This character is of great importance because the analysis results can serve as useful internal standards to correlate each other. In 2017, a study by Chen et al. described the use of the fluorescence and phosphorescence of CD powders for dual-channel detection of temperatures (Figure 9(d)) [91]. In their study, they found that the fluorescence and phosphorescence showed a linear and a double-exponential decay behaviors toward the decrease of temperature in the region from 90 to 30°C (Figure 9(e)), respectively. These results imply a different action mechanism of temperature on the dual-mode emission of CDs.

A development in dual-channel sensing of pH was recently reported by Su and coworkers [51], who demonstrated that deprotonation of P–O bonds and aggregation of CDs causing by alkalinity increase can simultaneously lead to the decrease in the intensity of the dual-mode emissions (Figures 9(f)–9(i)). In comparison, the phosphorescence component showed a wider linear pH response region as compared to fluorescence. In a follow-up work [95], they extended this strategy for the sensing of tetracycline based on an inner filter effect of the absorbed tetracycline molecules on the dual-mode emission of CDs. The detection limits from fluorescence and phosphorescence mode were estimated to be 5.18 and 12.4 nmol L^−1^, respectively.

Besides, afterglow CDs have been proven effective in the sensing of O_2_ due to the fact that the excited triplet species of afterglow CDs are highly susceptible to O_2_. In one such report, Bai and coworkers recently described the use of afterglow composites of CDs@MgAl-LDHs to realize the quantitative probing of O_2_ in a large concentration range from 0 (pure N_2_) to 100 (v%) [83]. The basic sensing mechanism relies on the deactivation of the excited triplet species by conversion of O_2_ from triplet to singlet through energy transfer. These results also suggest the promising use of afterglow CDs in the field of photodynamic therapy because the *in situ* formed singlet O_2_ molecules are toxic toward cancer cells [109].

### 5.2. Bioimaging

**Figure 10:**
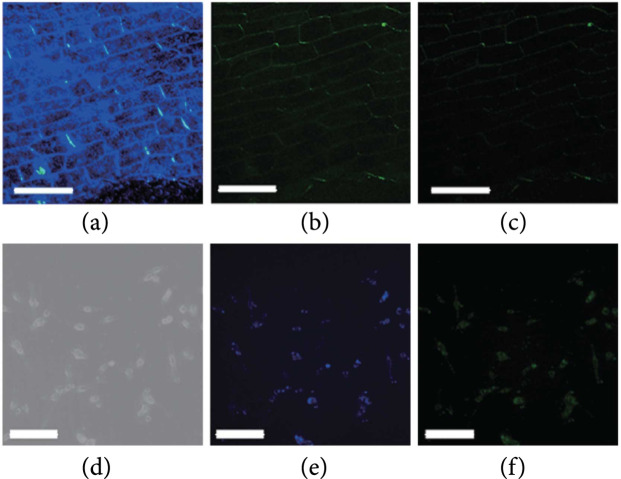
Fluorescence (a) and phosphorescence (b, c) images of CDs@SiO_2_-treated onion bulb epidermal tissue. Note that the delay times for (b) and (c) are 0.5 and 1 s, respectively. Scale bar: 200 *μ*m. (d–f) Bright-field, fluorescence, and phosphorescence images of CDs@ SiO_2_-treated mouse breast carcinoma EM-6 cells. The delay time for (f) is 1 s. Scale bar: 50 *μ*m (adapted and copyright permission [46] (a–f), American Chemical Society).

The use of afterglow CDs for bioimaging is also governed by the consideration of reducing background autofluorescence of tissues. In 2019, Li et al. first reported the use of CDs@SiO_2_ nanocomposites for imaging onion bulb epidermal tissue [46]. The results show that the outline of the onion bulb epidermal cell walls is changed from blue to green when the UV excitation was ceased (Figures 10(a)–10(c)). Thereafter, they further realized imaging of EM-6 mouse breast carcinoma cells by taking advantage of the long lifetime of the phosphorescence of the CDs@SiO_2_ nanocomposites (Figures 10(d)–10(f)). Despite these advances, widespread application of afterglow CDs in bioimaging needs to overcome the challenge that their excitation is mainly limited to UV light. UV light has a low penetration depth and shows a strong scattering issue, largely decreasing the usefulness of afterglow CDs in the application of bioimaging [110]. One solution is likely to extend the excitation wavelength of afterglow CDs to the visible region via surface composition control. Alternatively, the excitation wavelength may be further shifted to the near-infrared (NIR) region by integrating afterglow CDs with lanthanide-doped upconversion nanoparticles [111]. In the later design, NIR irradiation is first converted to UV or visible photons, serving as a secondary excitation source to excite afterglow CDs [112].

### 5.3. Anticounterfeiting

Since the report of their unique optical attributes comprising a long-lived phosphorescence and a short-lived fluorescence in 2013, afterglow CDs or CD-based nanocomposites have been considered one of the most promising classes of optical materials for anticounterfeiting [25]. The long-lived emission, which can be visualized by the naked eyes after ceasing excitation, shows the capability of adding an additional time-domain color code for information encoding without leading to additional complexity in authentication [6]. The tunable nature of the afterglow luminescence further extends the versatility of CDs in the application in this field via enhancing information storage strength.

**Figure 11:**
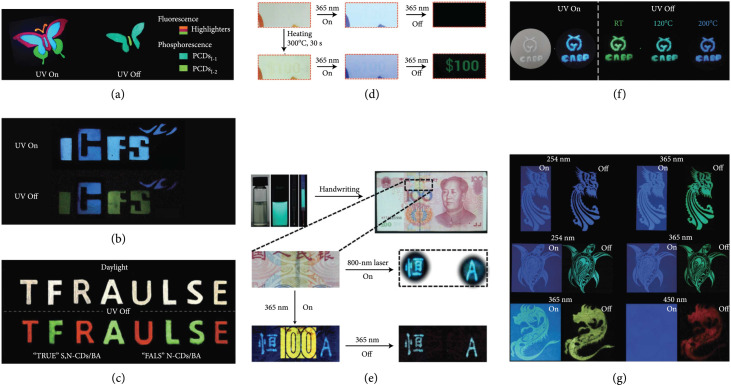
(a–c) Anticounterfeiting application of afterglow CDs on the basis of the long-lived and color-tunable attributes of their afterglow luminescence (adapted and copyright permission [45] (a), Wiley-VCH Verlag GmbH & Co. KGaA; [57] (b), American Chemical Society; [39] (c) Wiley-VCH Verlag GmbH & Co. KGaA). (d) Anticounterfeiting on the basis of heating-assisted conversion of fluorescence CDs to phosphorescence CDs (adapted and copyright permission [76], Wiley-VCH Verlag GmbH & Co. KGaA). (e) Anticounterfeiting based on multimode emission of CDs, namely, fluorescence, upconversion emission, and phosphorescence (adapted and copyright permission [50], Wiley-VCH Verlag GmbH & Co. KGaA). (f, g) Anticounterfeiting based on the use of temperature- and excitation-dependent afterglow luminescence of CDs (adapted and copyright permission [97] (f) and [44] (g), Royal Society of Chemistry).

In the early stage, considerable efforts have been devoted to realizing color tuning of the long-lived afterglow emission of CDs, thereby demonstrating their feasibility of anticounterfeiting application. The anticounterfeiting mechanism is described as follows: upon UV excitation, a mixed fluorescence and afterglow emission was presented, while a pure encoded afterglow emission appeared when ceasing the UV excitation. In one such example, Tao and coworkers patterned a covert butterfly with the use of green afterglow CDs and commercial fluorescent materials as inks on paper [45]. Under UV illumination, the two components were both excited, rendering a colorful butterfly pattern. Upon ceasing the excitation, only the encrypted green RTP pattern presented and lasted for a few seconds (Figure 11(a)). In a parallel effort, Liu et al. reported the observation of blue TADF and green RTP from CDs@zeolite nanocomposites and further demonstrated their anticounterfeiting application on the basis of the luminescence change of the patterns before and after the termination of the excitation (Figure 11(b)) [28, 57]. A recent study by Li et al. demonstrated that multicolor long-lived emissions can be obtained via the choice of appropriate CDs in the preparation of CD-boric acid (BA) composites [39]. Of particular interest is the generation of red phosphorescence (emission peak at 570 nm) via embedding S,N-doped CDs into the matrices (Figure 11(c)). These results suggest the success of creating three primary long-lived red, green, and blue emissions under excitation at a single excitation wavelength (365 nm), allowing the feasibility of fine-tuning the afterglow color output of the covert pattern via the use of a mixture of the afterglow CDs.

Notably, the development of stimulus-responsive afterglow CDs is particularly attractive for anticounterfeiting due to their improved capability in data encoding. In 2018, Jiang et al. reported a facile and quick pathway to convert fluorescent CDs to phosphorescent CDs through heating and argued that the CDs can be used as a new class of heat-responsive ink for anticounterfeiting [76]. In their demonstration, prior to heating treatment, a covert pattern of “$100” on a coupon cannot be visualized under UV light illumination due to the presence of strong background fluorescence (Figure 11(d)). In comparison, after being treated with a heat gun (300°C, 30 s), the covert pattern appears in green after terminating the excitation. However, the practical utility of such heat-responsive CDs for high-level anticounterfeiting is limited by their irreversible conversion from phosphorescent to fluorescent after the heating treatment.

Afterglow CDs with additional modes of emission are reported with the high suitability for multilevel anticounterfeiting applications. The observation of triple-mode emissions of CDs was first reported in 2016 by Jiang et al., who further demonstrated the potential application of such CDs for triple-level banknote anticounterfeiting [50]. In their results, we first created patterns of a Chinese character “heng” and an English letter “A” by printing on a banknote. They then obtained trimode optical anticounterfeiting attributes, including blue fluorescent patterns upon excitation at 365 nm, cyan upconversion features under irradiation with a femtosecond pulse laser (800 nm), and afterglow luminescent patterns upon ceasing the UV excitation (Figure 11(e)).

The dependence of the afterglow luminescence of CDs on temperature and excitation wavelength can also be harnessed to fabricate multiple color codes for anticounterfeiting [84, 97]. In 2020, Sun et al. made a pattern with the use of CDs@SiO_2_ as security ink and found that the pattern is presented in bright blue under excitation at 365 nm (Figure 11(f)) [97]. Once the excitation was terminated, a color transition of the pattern from blue to green was observed, and the resulting green afterglow pattern can last for a few seconds. More interestingly, the afterglow pattern can undergo from green to cyan and to blue when enhancing temperature from RT to 120 and to 200°C, respectively. In a parallel work, Lin et al. have prepared three different types of N-doped CD-biuret@urea composites that have excitation wavelength-dependent afterglow luminescence. These composites were then used as security inks to make a variety of patterns that have the ability to change from blue or cyan (top panel) and cyan to green (middle panel) and from yellow to red (bottom panel), respectively, upon ceasing the excitation at different wavelengths. Specifically, the former two optical scenarios were observed with the change of excitation wavelength from 254 to 365 nm, while the latter case was observed by altering the excitation wavelength from 365 to 450 nm (Figure 11(g)) [44].

### 5.4. Data Encryption

**Figure 12:**
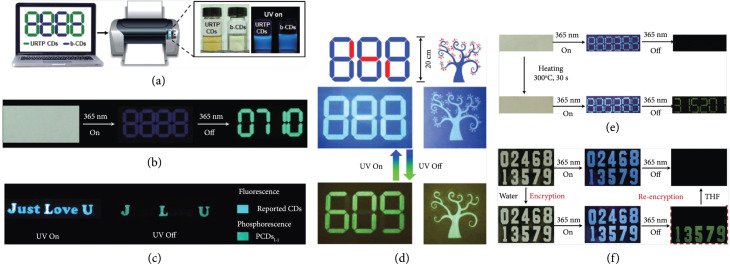
(a) Schematic representation of the creation of covert patterns via inkjet printing with the use of afterglow CDs as encoding ink. (b, c) Data encryption on the basis of the longer emission lifetime of the afterglow luminescence relative to the fluorescence of CDs (adapted and copyright permission [30, 45, 48], Wiley-VCH Verlag GmbH & Co. KGaA). (e) Data encryption based on the convertible nature of fluorescence CDs to phosphorescence CDs upon heating treatment (adapted and copyright permission [76], Wiley-VCH Verlag GmbH & Co. KGaA). (f) Advanced data encryption on the use of reversible aggregation-induced phosphorescence of CDs (adapted and copyright permission [40], Wiley-VCH Verlag GmbH & Co. KGaA).

Data encryption refers to decoding hidden information upon the use of additional stimulation. The underlying mechanism for data encryption is essentially identical to that for anticounterfeiting. On the basis of the above-mentioned designs for anticounterfeiting, a diversity of afterglow CDs has been proven effective for data encryption. In such data encryption applications, fluorescent probes and afterglow CDs were simultaneously used as inks to encode false and correct data via inkjet printing, respectively (Figure 12(a)). Taking advantage of the similar fluorescence emissions of the two types of inks, the correct information encoded by afterglow CDs was hidden with the false information upon UV excitation. However, the correct information appears and is separated from the interfering patterns upon ceasing the UV excitation. For example, by making use of afterglow CDs as inks, the groups of Jiang et al., Tao et al., and Long et al. have successfully encrypted the data “0710,” “JLU,” “609,” and “a bare tree” in the blue fluorescence interfering information and then decrypted the data upon the ceasing the UV excitation benefiting from the long emission lifetime of the afterglow luminescence (Figures 12(b)–12(d)) [30, 45, 48].

Similarly, Jiang and coworkers have extended the heat-responsive CDs from anticounterfeiting to data encryption. In this case, the encrypted data of “315201” only be visualized upon heating treatment followed by ceasing UV excitation (Figure 12(e)). The limitation of the irreversible transformation of the CDs from fluorescent to phosphorescent through the heating treatment can be overcome by their recent work on aggregation-induced RTP [40]. In the follow-up study, they showed that the hidden data of “13579” only appeared when the data were first treated with water followed by turning off of UV irradiation (Figure 12(f)). More importantly, the observed data can be further encrypted by THF wetting to destroy the aggregation state of the CDs. Obviously, the aggregation-induced RTP can provide new opportunities and flexibility of CDs for practical data encryption. However, control over the wetting degree of the encrypted data to destroy the aggregation-induced RTP while keeping the shape of the patterns may present as a new challenge.

## 6. Conclusions and Outlook

This review has summarized the recent advances in the field of afterglow CDs ranging from physical fundamentals, afterglow activation strategy, and emergent afterglow luminescence properties to multiple applications. Owing to their intriguing optical properties and widespread potential applications, afterglow CDs should continue to be a focus of a growing body of research in materials science and optoelectronics. Prior to the realization of the full practical utility of afterglow CDs, a cooperative and coordinated effort from multidiscipline is necessary to address the following challenges:

(i) Improving the afterglow efficiency and lifetime of CDs. Improvement of the optical attributes, including efficiency and lifetime, is a fundamental challenge for afterglow CDs. Currently, doping of heteroatoms, such as N and P, has proven effective in enhancing the probability of the occurrence of intersystem crossing into CDs to elevate the afterglow efficiency. The realization of such doping mainly relies on the selection of appropriate heteroatom-containing precursors in CD preparation. Additional measures are needed to stabilize the triplet excited states of CDs to enhance the afterglow lifetime of CDs by taking advantage of covalent bonding, hydrogen bonding, and other fixation interactions
(ii) Providing a better understanding of the origins of the afterglow luminescence of CDs. At present, the afterglow luminescence of CDs was studied from their ensembles either in the solution or in the solid state. Such ensemble measurements cannot provide convincing data to explain the unique afterglow attributes of CDs, such as excitation-dependent afterglow luminescence. A much-needed solution is to carry out afterglow characterization at the single-particle level to exclude the interference from other neighboring CDs and impurities. The poor understanding of the afterglow mechanism is also because of the difficulty in revealing the detailed structure of the CDs. Cross-linked polymer chains immobilized on the surface of CDs that seem to be essential to the afterglow luminescence of CDs are invisible in conventional TEM inspection. Detailed characterization of the surface moieties at the atomic level is highly necessary for a deep understanding of the afterglow mechanism
(iii) Extending the afterglow luminescence to the red spectral region. The afterglow luminescence of the majority of CDs is in the region of 500-540 nm (green emission); extending the afterglow emission to a wavelength longer than 600 nm remains a formidable challenge. Two encouraging reports suggest that enhancing synthetic temperatures may be applicable to the synthesis of CDs with red afterglow luminescence via exquisite control of their surface oxidation states. Alternatively, doping of red emissive metal ions, such as Mn^2+^ and Eu^3+^, into the protective matrices of afterglow CDs is likely to be an indirect pathway to produce red afterglow luminescence of CD-based materials via energy transfer from the CDs to the doped ions
(iv) Enhancing the stability of the afterglow luminescence of CDs. Practical use of afterglow CDs needs their afterglow luminescence to show excellent tolerance toward the water, M^n+^, and organic solvents. However, the majority of the reported afterglow CDs or their composites are unable to meet these requirements. Recent research progress suggests the promise of using SiO_2_ encapsulation to address the concern of optical instability. However, the conventional Stöber method shows the inability to prepare well-dispersed CDs@SiO_2_ nanoparticles probably due to the complex surface chemistry of the afterglow CD cores. Special efforts are necessary to refine the SiO_2_ encapsulation method to gain remarkable controllability of the size and morphology of the resulting CD@SiO_2_ nanocomposites. Alternatively, utilization of preformed mesoporous SiO_2_ nanoparticles to load with CDs followed by growth of a protective SiO_2_ shell may provide a much-needed solution to address this challenge
(v) Exploring efficient synthetic and purification strategies for afterglow CDs. Afterglow CDs are often prepared by carbonization of polymer and molecule precursors under microwave irradiation or hydrothermal conditions. Considerable amounts of the precursors were converted to emissive oligomers or nonemissive big-sized carbon aggregates. The carbonization conditions need to be further elaborated to enhance the yield of afterglow CDs. In addition, in the purification step, the synthetic mixture was often subjected to dialysis to remove precursor oligomers and inorganic salts after the removal of big-sized carbon aggregates. This is time-consuming and unsuitable for large-scale purification. Physical absorption by mesoporous carbon materials followed by centrifugation may be applied to accelerate the purification efficiency of afterglow CDs
